# Supplementary material for: Identification of Novel Gene Cluster Potentially Associated with Insecticide Resistance in Anopheles gambiae s.l
Source: Genes (Basel). 2025 Aug 28;16(9):1018. doi: 10.3390/genes16091018 (PMC12470184; doi:10.3390/genes16091018)
Supplement: Supplementary file 1 [file genes-16-01018-s001.zip › Figure S1.docx]

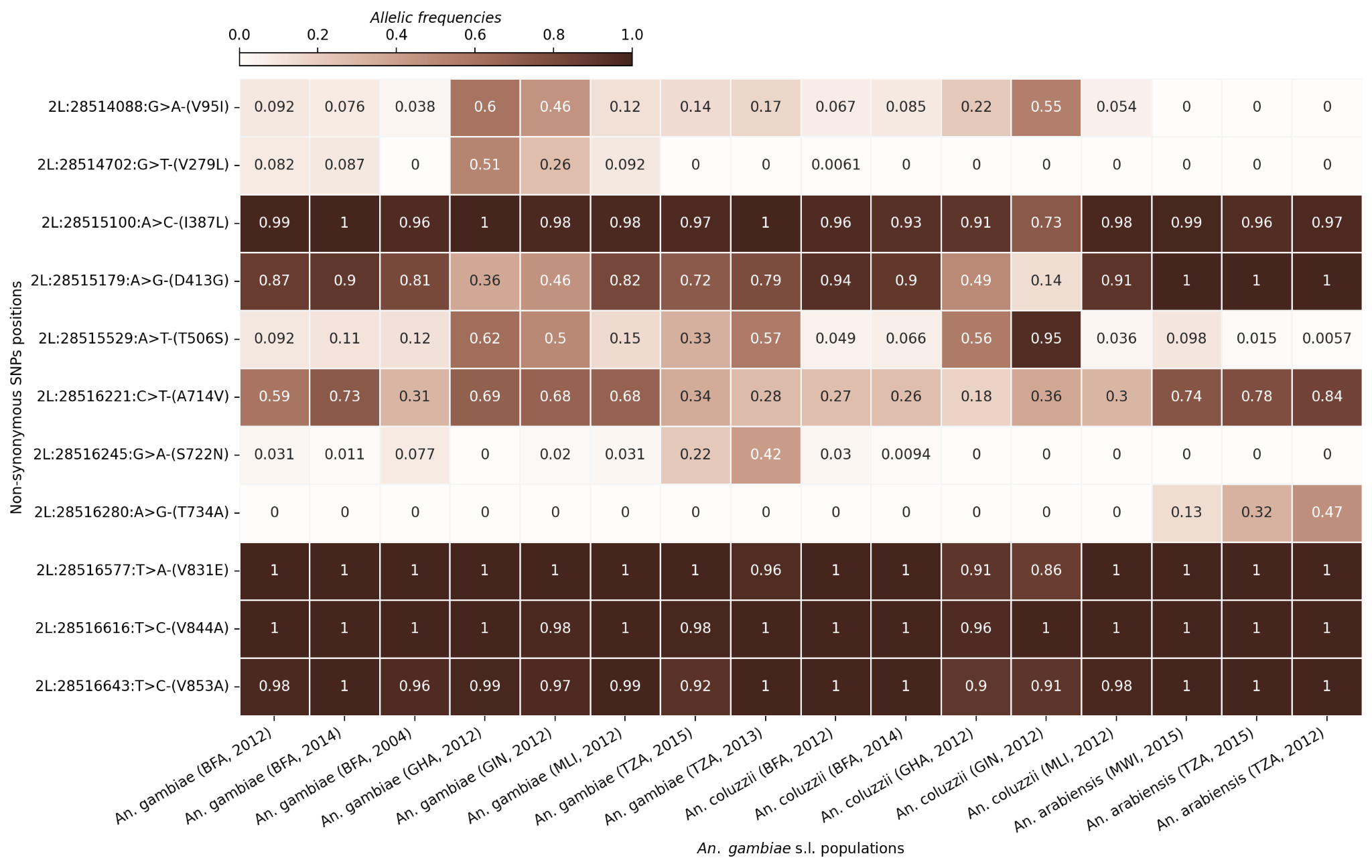


**Figure S1**. SNP allele frequencies in the *AGAP006220* gene in the different *An. gambiae s.l*. populations. Results were filtered to just non-synonymous SNPs that are at frequency above 40% in at least one cohort. The X‑axis shows the different cohorts (country, species and sampling period). The Y‑axis shows the SNPs positions in the gene and the corresponding amino acid change. The gradient color bar shows the distribution of the allelic frequencies.
