# Supplementary material for: Identification of Novel Gene Cluster Potentially Associated with Insecticide Resistance in Anopheles gambiae s.l
Source: Genes (Basel). 2025 Aug 28;16(9):1018. doi: 10.3390/genes16091018 (PMC12470184; doi:10.3390/genes16091018)
Supplement: Supplementary file 1 [file genes-16-01018-s001.zip › Figure S2.docx]

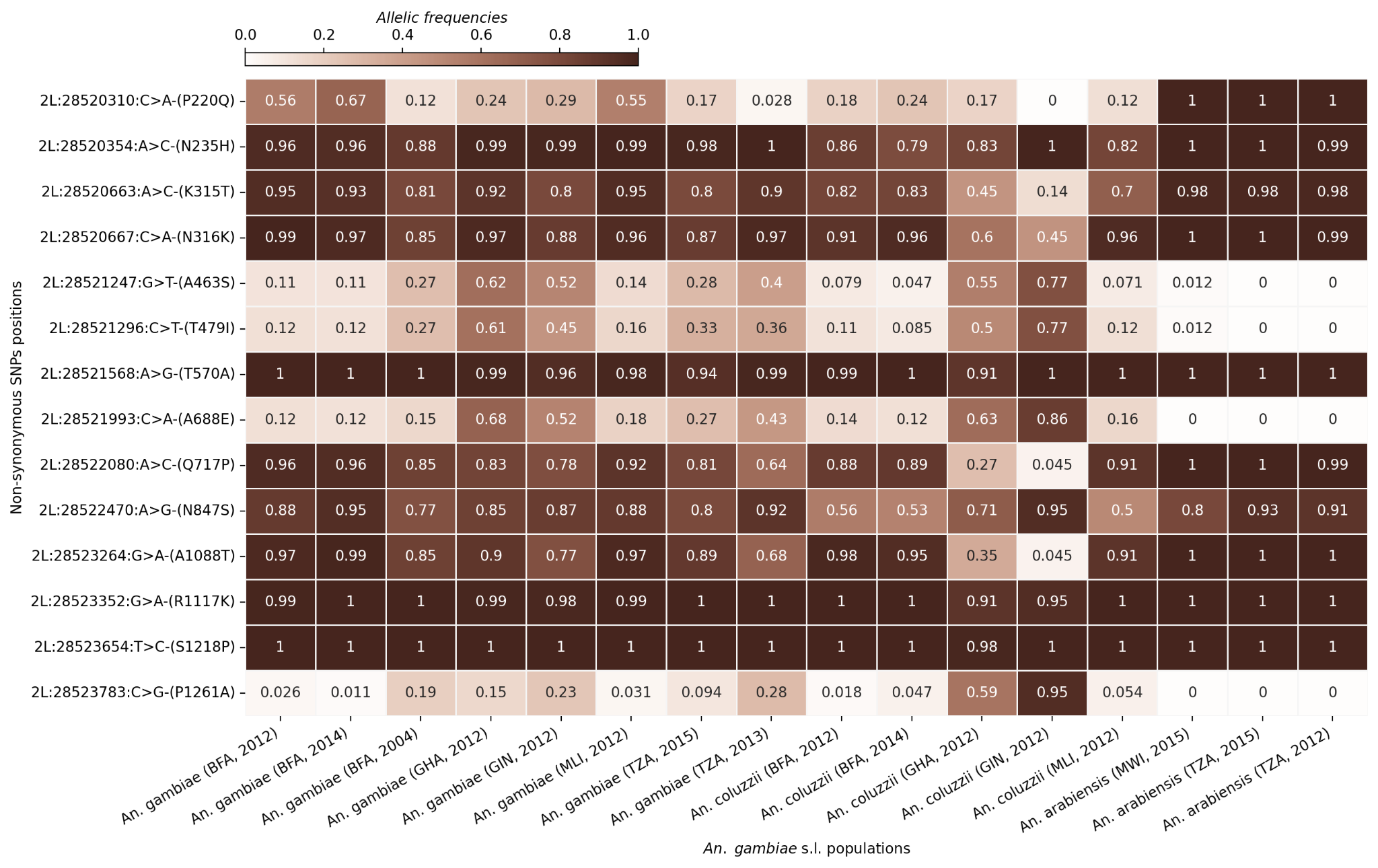


**Figure S2**. SNP allele frequencies in the *AGAP006221* gene in the different *An. gambiae s.l*. populations. Results were filtered to just non-synonymous SNPs that are at frequency above 75% in at least one cohort.
