# Supplementary material for: Identification of Novel Gene Cluster Potentially Associated with Insecticide Resistance in Anopheles gambiae s.l
Source: Genes (Basel). 2025 Aug 28;16(9):1018. doi: 10.3390/genes16091018 (PMC12470184; doi:10.3390/genes16091018)
Supplement: Supplementary file 1 [file genes-16-01018-s001.zip › Table S1.docx]

**Table S1.** Study sites, collection year and species composition

| Country | Year | Species | | |
| --- | --- | --- | --- | --- |
|  |  | *An. gambiae s.s.* | *An. coluzzii* | *An. arabiensis* |
| Angola | 2009 | 0 | 81 | 0 |
| Burkina Faso | 2004 | 13 | 0 | 0 |
|  | 2012 | 89 | 82 | 0 |
|  | 2014 | 46 | 53 | 3 |
| Democratic Republic of the Congo | 2015 | 76 | 0 | 0 |
| Cote d'Ivoire | 2012 | 0 | 80 | 0 |
| Cameroon | 2005 | 90 | 7 | 0 |
|  | 2009 | 303 | 0 | 0 |
|  | 2013 | 23 | 19 | 2 |
| Ghana | 2012 | 36 | 64 | 0 |
| Guinea | 2012 | 84 | 7 | 0 |
| Mali | 2004 | 33 | 36 | 2 |
|  | 2014 | 33 | 27 | 0 |
| Malawi | 2015 | 0 | 0 | 41 |
| Tanzania | 2012 | 0 | 0 | 87 |
|  | 2013 | 32 | 0 | 1 |
|  | 2015 | 32 | 0 | 137 |
| Uganda | 2012 | 207 | 0 | 82 |
